# Case report: remedial microdissection testicular sperm extraction after onco-microdissection testicular sperm extraction failure

**DOI:** 10.1097/MD.0000000000037201

**Published:** 2024-02-23

**Authors:** Yi Zheng, Ding-Ming Li, Fu-Ping Li, Xiao-Hui Jiang, Luo Yang, Rui Qu, Heng-Zhou Bai, Gui-Cheng Zhao, Kun Tian

**Affiliations:** a Human Sperm Bank, West China Second University Hospital of Sichuan University, Chengdu, Sichuan 610041, China; b Department of Andrology, West China Second University Hospital of Sichuan University, Chengdu, Sichuan 610041, China; c Key Laboratory of Birth Defects and Related Diseases of Women and Children (Sichuan University), Ministry of Education, Chengdu, Sichuan 610041, China; d Department of Urology, West China School of Public Health and West China Fourth Hospital, Sichuan University, Chengdu, Sichuan 610000, China

**Keywords:** azoospermia, case report, salvage therapy, testicular cancer, testicular sperm extraction

## Abstract

**Background:**

Testicular cancer (TC) mostly occurs in men aged 14 to 44. Studies have shown that TC seriously damages male fertility, and 6% to 24% of patients with TC were even found to suffer from azoospermia when they are diagnosed. At present, some studies have pointed out that onco-microdissection testicular sperm extraction (mTESE) can extract sperm from tumor testicles. However, there are almost no reports on remedial measures after onco-mTESE failure. Given the valuable opportunity for fertility preservation in patients with TC and azoospermia, it is necessary to provide effective remedial methods for patients with failed onco-mTESE.

**Methods:**

Two young men, who were diagnosed with TC and also found to have azoospermia, tried onco-mTESE while undergoing radical orchiectomy for fertility preservation. However, sperm extraction failed in both patients. Subsequently, the isolated testicular tissue of the patient in case 1 suffered from TC again, and the patient in case 2 was scheduled to receive multiple cycles of gonadotoxic chemotherapy. Because both had a plan to have a birth in the future, we performed remedial mTESE.

**Results:**

Sperm was successfully extracted from both patients. The patient recovered well, without complications. The patient couple in case 1 underwent 1 intracytoplasmic sperm injection (ICSI) cycle but did not achieve clinical pregnancy.

**Conclusions:**

There is still an opportunity to extract sperm successfully using onco-mTESE, despite the difficulty of fertility preservation in TC patients with azoospermia. If sperm extraction from the tumor testis fails, implementing remedial mTESE as early as possible would likely preserve the last chance of fertility for these patients.

## 1. Introduction

Testicular cancer (TC) accounts for 1% of newly diagnosed malignancies in men and mostly occurs in young men aged 14 to 44.^[1]^ Although the incidence of TC has increased in recent decades,^[2]^ the cure rate after treatment remains high, with an overall long-term survival of 97%.^[3]^ This means that a considerable number of patients still have fertility requirements during their survival. However, studies have shown that TC damages male fertility seriously,^[4–6]^ and 6% to 24% of patients with TC were found to suffer from azoospermia when they were diagnosed.^[7]^ Meanwhile, the treatment of TC is usually radical orchiectomy, after which approximately 50% of patients continue to receive chemotherapy.^[8]^ The most widely used chemotherapy regimens are bleomycin, etoposide, and cisplatin (BEP), which are considered moderately toxic to reproduction.^[9]^ Either radical orchiectomy or chemotherapy can damage a patient’s already impaired fertility.^[10]^ Therefore, preservation of fertility in these patients is particularly important. In most cases, clinicians try to cryopreserve sperm in semen before TC treatment; however, if a TC patient has confirmed azoospermia, it would be a huge challenge.

Fortunately, the use of microsurgical techniques has made it possible to obtain testicular sperm under difficult conditions, and it is worth trying in TC patients with azoospermia. Theoretically, in TC patients, microdissection testicular sperm extraction (mTESE) could more clearly identify the boundary between tumor tissue and normal tissue in the tumor testis, so as to extract seminiferous tubules that might contain spermatozoa in an area as far away from the tumor as possible. It can be performed in conjunction with a radical orchiectomy. The first case of onco-mTESE was reported by Binsaleh et al.^[11]^ There have been several case reports of successful sperm retrieval using onco-mTESE in the following decade. However, limited by the very small number of cases, there is still a lack of research with a high level of evidence to confirm the success rate of sperm extraction using this method. This means that a considerable number of TC patients may still face the dilemma of failed sperm extraction. However, when onco-mTESE fails, the timing of sperm extraction from the contralateral testis is still debated.

This case report presents the experience of 2 TC patients who underwent contralateral testicular sperm extraction after an initial failed onco-mTESE. Moreover, we attempted to discuss the risk of onco-mTESE failure and timing of remedial mTESE in the contralateral testis.

## 2. Case presentation

**Figure 1.**
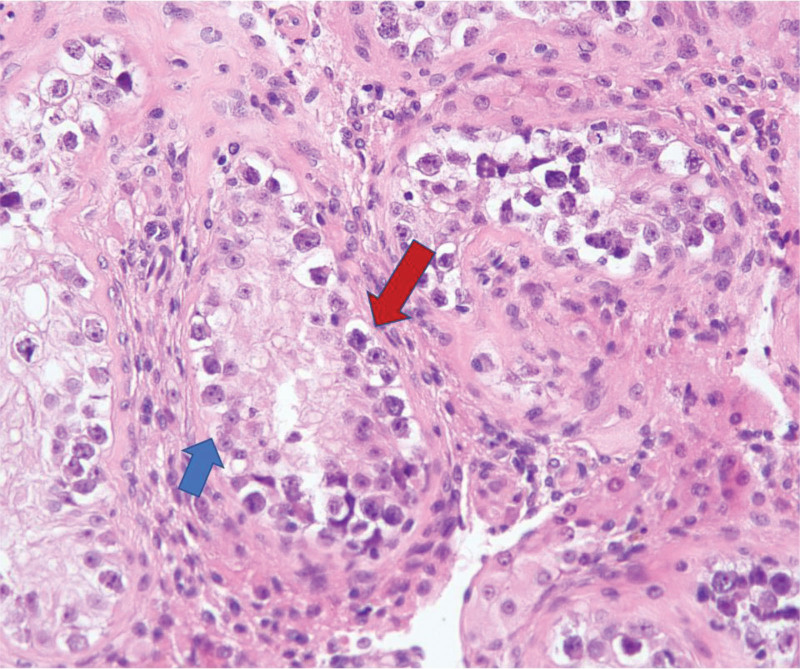
Histopathological findings of the resected testicular tumor. Hematoxylin–eosin stain of seminoma with seminiferous tubules surrounding the tumor (200×). Red arrow indicated the tumor cells and Sertoli cells were indicated by blue arrow.

Case one: A 34-year-old man presented with a primary infertility. The patient was otherwise healthy, apart from a left testicular mass on physical examination. The patient was found to have azoospermia after 2 semen analyses. His sex hormone levels were normal, karyotype was 46, XY, and there was no Y chromosome microdeletion. Ultrasonography of the testis confirmed the presence of a tumor approximately 4 cm in diameter in the left testis. Serum tumor marker tests revealed an elevated human chorionic gonadotropin (hCG) was 116.9 mIU/mL, alpha-fetoprotein (AFP) was 1.6 ng/mL, and lactate dehydrogenase (LDH) was 219 U/L. Computed Tomography scans of the chest, abdomen, and pelvis was normal. We planned to perform left radical orchiectomy in this patient. As the patient couple hoped to use intracytoplasmic sperm injection (ICSI) for assisted reproduction in the future, we decided to attempt onco-mTESE to extract and cryopreserve sperm from the left testis. An inguinal incision was made to mobilize and clamp the left spermatic cord intraoperatively, and the left testicle was excised. The tunica albuginea was opened under a 20 × microscope, and a lard-like tumor with a diameter of approximately 4.5 cm was found occupying most of the testicles. The seminiferous tubules were extracted several times in an area at least 5 mm away from the tumor edge, but no sperm was found. The final pathological evaluation revealed a seminoma with a germ cell hypoplasia/Sertoli cell-only status in the seminiferous tubules surrounding the tumor (Fig. 1). The TC stage group was classified as having stage IA.

**Figure 2.**
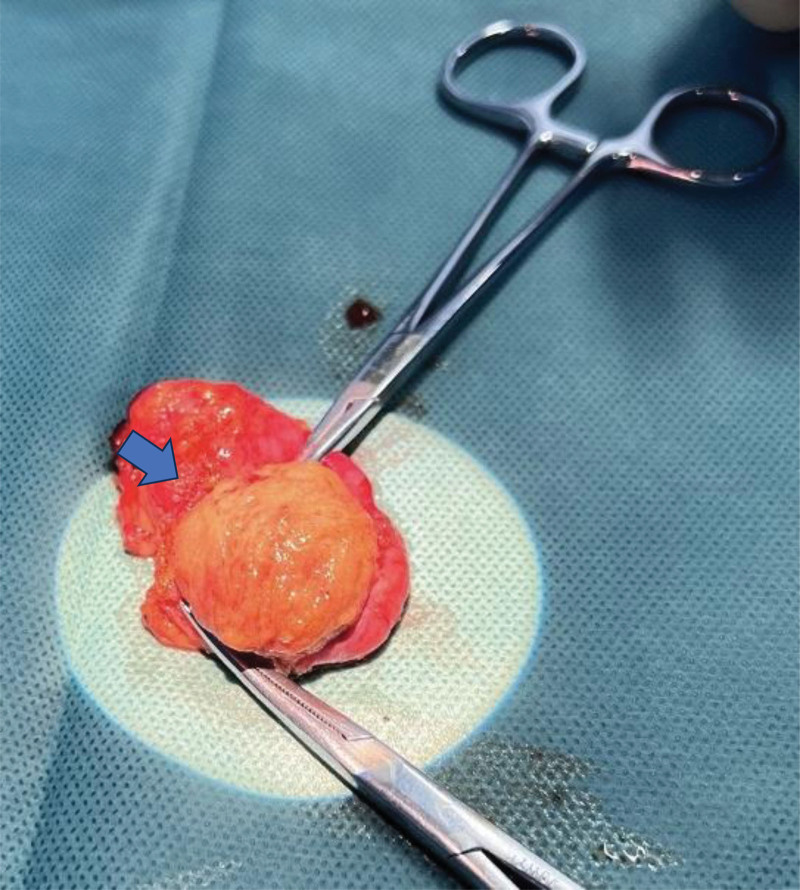
Macroscopic view of the testicular tumor after opening the tunica albuginea. Normal seminiferous tubules were visible in the surrounding area (indicated by blue arrows).

**Figure 3.**
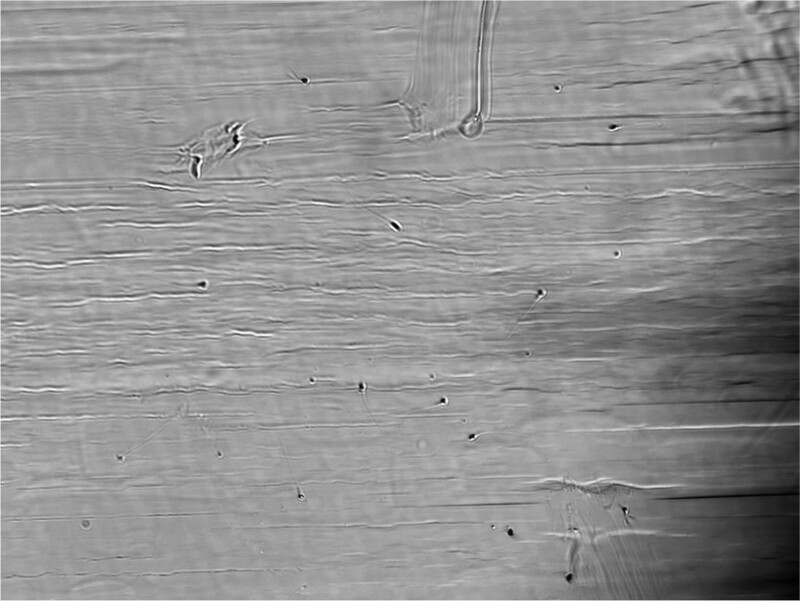
Some active spermatozoa with normal morphology were seen under high magnification microscopy.

**Figure 4.**
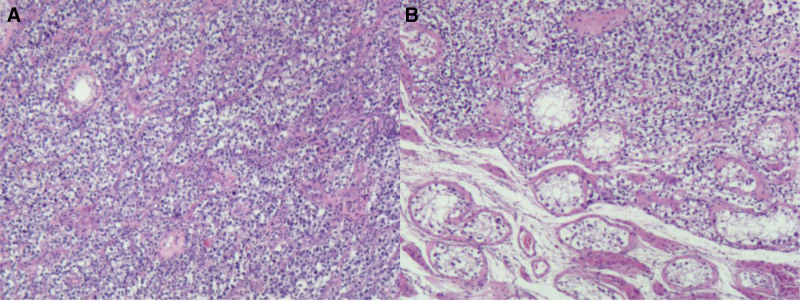
Histopathological findings of the resected testicular. Hematoxylin–eosin stain of seminoma (A) and seminiferous tubules surrounding the tumor with all levels spermatogenic cells and more spermatozoa (B) (100×).

The patient had an uneventful postoperative recovery and underwent active surveillance including regular testicular ultrasound, serum tumor marker detection, and clinical evaluation without chemotherapy. Sperm extraction was not attempted on the right testis immediately because the patient’s fertility program was delayed owing to the disease. 1 year after surgery, testicular ultrasonography revealed a tumor with a diameter of approximately 2 cm in the right testis. Serum tumor marker detection showed hCG was 78.9 mIU/mL, and AFP was 1.2 ng/mL. Sex hormone detection showed follicle-stimulating hormone was 3.8 IU/L, luteinizing hormone was 0.8 IU/L, testosterone (T) was 1.71 ng/mL, estradiol (E2) was 37.6 pg/mL. The patient was scheduled to undergo a right radical orchiectomy. Considering that the patient and his wife still planned for ICSI, onco-mTESE was performed on the right testis, and thick and plump seminiferous tubules were found and extracted at a distance >2 cm from the tumor edge (Fig. 2). Laboratory biologists detected mature spermatozoa under a phase-contrast microscope and cryopreserved them (Fig. 3). Postoperative pathological analysis showed that the tumor was a seminoma, spermatogenic cells of various levels and spermatozoa could be seen around the tumor, and the arrangement of cells of various levels in some lumens was slightly disordered (Fig. 4). The Johnsen scores were 9. The patient experienced no complications. After the surgery, the patient underwent active surveillance and did not receive chemotherapy. During the follow-up of 1 year, no signs of tumor recurrence were found, and the patient received continuous testosterone replacement therapy owing to the decline in testosterone levels.

The patient’s wife was a healthy 33-year-old woman who subsequently underwent ICSI. Eleven oocytes were obtained from the woman, 9 of which were at stage MII. The cryopreserved testicular tissue suspension was thawed, sperm with good morphology was selected, and 9 oocytes were injected with sperm. Then we obtained 5 embryos. 2 high-quality embryos were transferred and the rest were cryopreserved. Unfortunately, none of the transferred embryos achieved clinical pregnancy.

**Figure 5.**
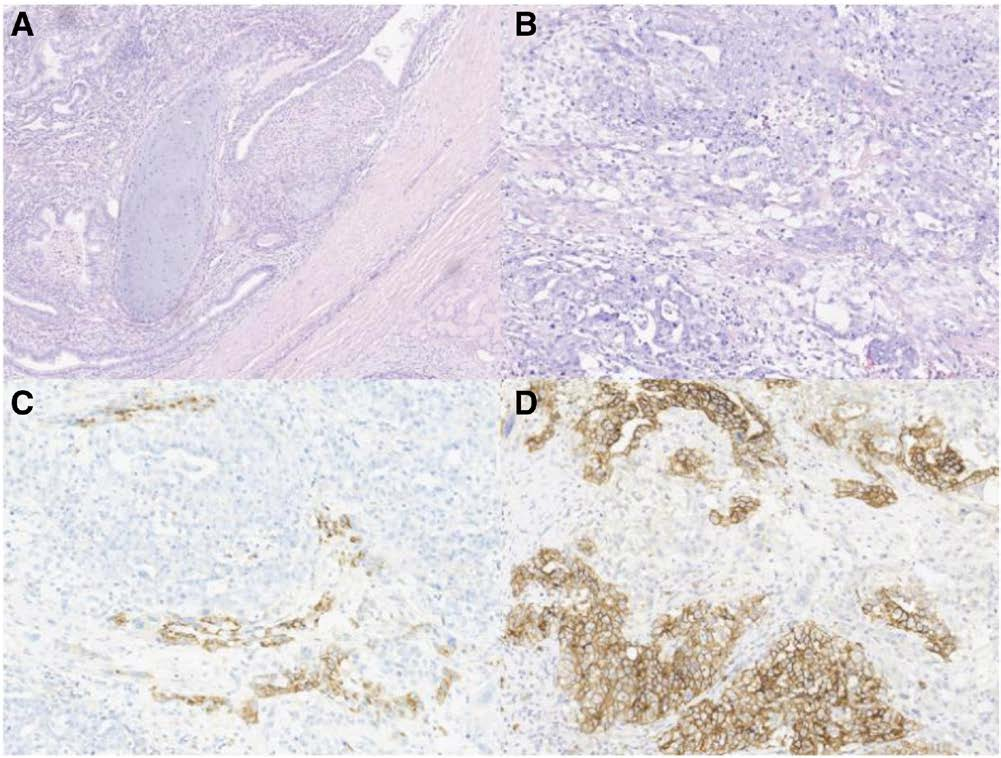
Histopathological findings of the resected testicular tumor. Hematoxylin–eosin stain of the immature teratoma (40×) (A) and embryonal carcinoma and yolk sac tumor (100×) (B). Immunohistochemical marker AFP positive (C) (100×). Immunohistochemical marker CD30 positive (D) (100×).

Case two: A 20-year-old male with no partner or childbirth. He had bilateral cryptorchidism during childhood. The patient was admitted to the urology center of another hospital because of the discovery of a mass in the left testis. Testicular ultrasonography and magnetic resonance imaging evaluated the volume of the left testis to be about 40 mL, in which the tumor was 3.9 × 6.3 × 2.5 cm (31.9 mL), and the right testis was found to be dysplasia (5.6 ml). Serum sex hormone showed follicle-stimulating hormone 0.8 IU/L, luteinizing hormone 1.3 IU/L, T 4.49 ng/mL, E2 48.0 pg/mL. The karyotype was 46, XY and no Y chromosome microdeletions were observed. The serum tumor marker levels showed HCG 853 mIU/ml, AFP 937.30 ng/ml, LDH 679 U/L. Other imaging findings revealed several solid nodules in both lungs and multiple enlarged lymph nodes in both groins. The urologists planned to perform left radical orchiectomy. Considering that the patient had not yet given birth, the urologist suggested that him sought advice from a center with fertility preservation conditions before tumor treatment. Therefore, the patient was referred to our center, and no sperm were found after 2 semen analyses. We then decided to use onco-mTESE to extract sperm from the left testis and cryopreserve it. An inguinal incision was made to mobilize and clamp the left spermatic cord intraoperatively, and the left testicle was excised. After opening the tunica albuginea under a 20 × microscope, a grayish-red tumor approximately 7 cm in diameter was found, occupying most of the testis, and no normal seminiferous tubules could be extracted. Postoperative pathological analysis revealed a malignant mixed germ cell tumor of the left testis (including embryonal carcinoma, yolk sac tumor, and teratoma), and no normal seminiferous tubules were found in the tissue surrounding the tumor (Fig. 5). Patients in the TC stage group of TC was stage IIIA.

**Figure 6.**
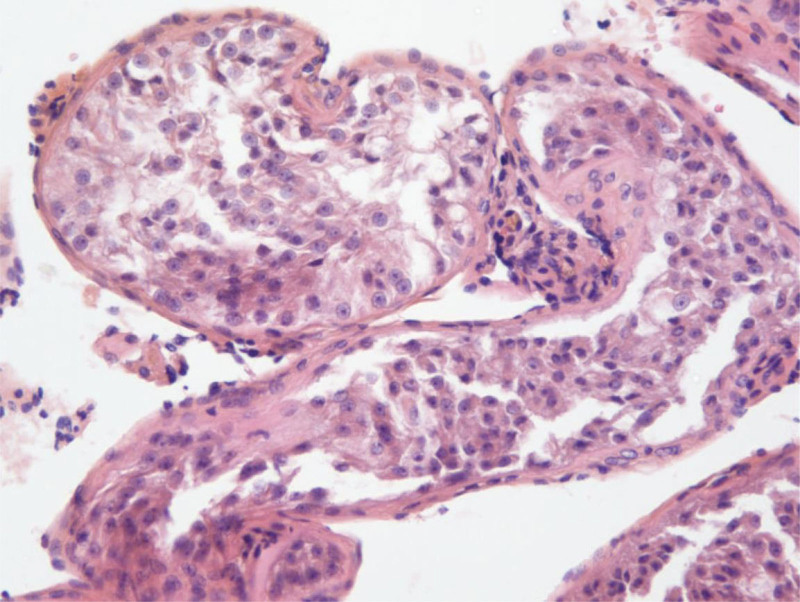
Histopathological findings of the right testicular tissue. Some of the seminiferous tubules were vitreous with only Sertoli cells (200×).

The patient was scheduled to receive multiple cycles of BEP chemotherapy after surgery; however, he still hoped to undergo ICSI-assisted reproduction in the future. We then performed remedial mTESE on the patient’s dysplastic right testis immediately before starting chemotherapy. During the surgery, it was found that the seminiferous tubules in the right testis were unequal in thickness, and relatively larger seminiferous tubules were extracted. Mature sperm were found in the laboratory and cryopreserved. Postoperative pathological analysis of randomly sampled testicular tissue showed germ cell hypoplasia/Sertoli cells only, and the Johnsen score was 2 (Fig. 6). The patient did not experience any surgical complications.

The patient then underwent 7 cycles of BEP chemotherapy, as planned. After treatment, the patient’s hCG and AFP levels continued to decrease. 6 months after the treatment, the patient’s chest and abdomen showed that the lung nodules and inguinal lymph nodes were significantly reduced. As the patient did not have a partner, cryopreserved sperm were not used.

## 3. Discussion

We report 2 rare cases in which the first attempts at onco-mTESE failed. We then performed remedial contralateral testicular mTESE (also onco-mTESE in case 1) because the isolated testicle of the patient in case 1 had suffered from TC again, and the patient in case 2 showed the characteristics of testicular dysgenesis syndrome (azoospermia, cryptorchidism, and TC)^[12,13]^ underwent reproductive toxic chemotherapy. Remedial mTESE was successful in both patients.

As TC can damage male fertility, fertility preservation is recommended in principle for all patients with TC. However, if TC patients are confirmed to have azoospermia during fertility assessment, the procedure of testicular sperm extraction should be put on the agenda. In 2000, Res et al^[14]^ reported successful TESE of healthy testes before the treatment of TC patients, and obtained live births through ICSI firstly. This makes it unnecessary for TC patients with azoospermia to wait for the end of treatment before attempting fertility preservation. Andrologists could thus attempt to advance the timing of sperm extraction before the potential fertility damage caused by TC treatment. Furthermore, for patients with bilateral testicular tumors and solitary testicular tumors, the issue of fertility preservation has begun to receive attention. Subsequently, successful sperm extraction from tumorous testes was reported.^[11,15]^ This has drawn attention to the spermatogenic function of tumorous testes. Retrospective studies of pathological samples from isolated tumor testes showed spermatogenesis in 62% to 80% of tumor testes, including patients with azoospermia.^[16,17]^ Therefore, azoospermic patients who lost the chance of fertility preservation before TC treatment could choose to undergo onco-mTESE. At the same time, this did not delay the timing of TC treatment and avoided damage to healthy testes. It is also the last choice for patients with bilateral testicular tumors and solitary testicular tumors. Finally, fertility preservation can still be attempted after the end of TC treatment in most cases. The study observed a small decrease in semen quality or risk of azoospermia after TC treatment,^[18,19]^ and in some cases increased.^[20]^ Sperm can be retrieved by TESE or mTESE, even in the presence of azoospermia.^[21]^

However, for TC patients with azoospermia, the situation was not as optimistic. Even with the implementation of onco-mTESE, more than 1/3 of the patients were still unable to extract sperm.^[7]^ Some studies have reported factors related to onco-TESE/mTESE outcomes, and the risk of sperm extraction failure increased when the tumor diameter was ≥4 cm and the seminiferous tubules were extracted <7.5 mm from the tumor margin.^[22,23]^ The results of our 2 cases were consistent with the conclusions of previous studies. However, options for subsequent fertility preservation in patients with failed onco-mTESE remain unclear. There is a paucity of studies on remedial sperm extraction after onco-TESE/mTESE failure. There was also a lack of discussion on the timing of the remedies at the same time. More recently, 2 case series suggested that when onco-mTESE failed to retrieve sperm, simultaneous sperm extraction from the healthy side could be performed and was successful in some patients.^[24,25]^ These cases could be considered as preventive remedies. In the reported cases, the choice of remedies was mainly determined by the patient’s subsequent condition. Fortunately, at the time of remedial mTESE, the spermatogenic function of the patient’s isolated testis had not yet failed due to tumors or testicular dysgenesis syndrome, but there is currently no method to predict the cutoff time for complete loss of spermatogenic function. Combined with our cases, the lesson was that when onco-mTESE failed, remedies should be performed on the contralateral testis as early as possible, even simultaneously. This was especially true when the patient was exposed to other risk factors impairing fertility, such as abnormal sex hormone levels, testicular dysgenesis syndrome, and multiple cycles of BEP chemotherapy. In addition, although bilateral testicular tumors are very rare, accounting for only 1.7% of all testicular tumors, 65% develop contralateral tumors after orchiectomy.^[26,27]^ This situation should still be considered; in fact, several case reports have noted this.^[15,28]^ Therefore, we believe that performing mTESE of the contralateral testis as early as possible after onco-mTESE failure could prevent patients from missing their last opportunity for fertility preservation.

Our case report described 1 failed ICSI cycle. Although 2 embryos were obtained, none of the subsequent embryo transfers were performed. Only a few cases of onco-mTESE have resulted in live births.^[24,29,30]^ Several case reports have described failed ICSI attempts.^[15,31,32]^ Due to the lack of detailed reports on these ICSIs and the small number of cases, larger studies are needed to compare the outcomes of assisted reproduction with sperm extracted from tumor testes vs healthy testes.

## 4. Conclusion

TC can seriously damage fertility and even lead to azoospermia, but these patients still had the opportunity to obtain sperm by onco-mTESE. If sperm extraction from the tumor testis fails, salvage mTESE of the contralateral testis is performed. Considering the risk of further spermatogenesis failure caused by reproductive toxicity chemotherapy, testicular dysgenesis syndrome, and contralateral testicular tumors, remedial mTESE should be implemented as early as possible, even concurrently with onco-mTESE.

## Author Contributions

YZ collected the relevant literature and was a major contributor to writing the manuscript. GCZ, RQ, and HZB were involved in the collection of clinical data. KT provides the collection and processing of the images. DML, LY, and FPL provided initial ideas and report design. XHJ revised the final manuscript and provided the recommendations. All authors have read and approved the final manuscript.

**Conceptualization:** Ding-Ming Li, Fu-ping Li, Luo Yang.

**Data curation:** Yi Zheng, Rui Qu, Heng-Zhou Bai, Gui-Cheng Zhao, Kun Tian

**Formal analysis:** Heng-Zhou Bai.

**Methodology:** Rui Qu.

**Project administration:** Fu-ping Li, Luo Yang.

**Software:** Gui-Cheng Zhao, Kun Tian.

**Supervision:** Xiao-Hui Jiang.

**Validation:** Ding-Ming Li, Fu-ping Li, Xiao-Hui Jiang.

**Writing—original draft:** Yi Zheng.

**Writing—review & editing:** Ding-Ming Li, Xiao-Hui Jiang.

## References

[1] Cheng L Albers P Berney DM. Testicular cancer. Nat Rev Dis Primers. 2018;4:29. 30291251 10.1038/s41572-018-0029-0

[2] Gurney JK Florio AA Znaor A. International trends in the incidence of testicular cancer: lessons from 35 years and 41 countries. Eur Urol. 2019;76:615–23. 31324498 10.1016/j.eururo.2019.07.002 PMC8653517

[3] Patrikidou A Cazzaniga W Berney D. European Association of Urology Guidelines on testicular cancer: 2023 update. Eur Urol. 2023;84:289–301. 37183161 10.1016/j.eururo.2023.04.010

[4] Jacobsen R Bostofte E Engholm G. Risk of testicular cancer in men with abnormal semen characteristics: cohort study. BMJ. 2000;321:789–92. 11009515 10.1136/bmj.321.7264.789 PMC27489

[5] Raman JD Nobert CF Goldstein M. Increased incidence of testicular cancer in men presenting with infertility and abnormal semen analysis. J Urol. 2005;174:1819–22; discussion 1822. 16217294 10.1097/01.ju.0000177491.98461.aa

[6] Mancini M Carmignani L Gazzano G. High prevalence of testicular cancer in azoospermic men without spermatogenesis. Hum Reprod. 2007;22:1042–6. 17220165 10.1093/humrep/del500

[7] Moody JA Ahmed K Yap T. Fertility managment in testicular cancer: the need to establish a standardized and evidence-based patient-centric pathway. BJU Int. 2019;123:160–72. 29920910 10.1111/bju.14455

[8] Guney S Guney N Sonmez NC. Risk-adapted management for patients with clinical stage I non-seminomatous germ cell tumour of the testis. Med Oncol. 2009;26:136–42. 18821067 10.1007/s12032-008-9095-6

[9] Dohle GR. Male infertility in cancer patients: review of the literature. Int J Urol. 2010;17:327–31. 20202000 10.1111/j.1442-2042.2010.02484.x

[10] Petersen PM Skakkebaek NE Vistisen K. Semen quality and reproductive hormones before orchiectomy in men with testicular cancer. J Clin Oncol. 1999;17:941–7. 10071288 10.1200/JCO.1999.17.3.941

[11] Binsaleh S Sircar K Chan PT. Feasibility of simultaneous testicular microdissection for sperm retrieval and ipsilateral testicular tumor resection in azoospermic men. J Androl. 2004;25:867–71. 15477357 10.1002/j.1939-4640.2004.tb03155.x

[12] Skakkebaek NE Rajpert-De Meyts E Main KM. Testicular dysgenesis syndrome: an increasingly common developmental disorder with environmental aspects. Hum Reprod. 2001;16:972–8. 11331648 10.1093/humrep/16.5.972

[13] Wohlfahrt-Veje C Main KM Skakkebaek NE. Testicular dysgenesis syndrome: foetal origin of adult reproductive problems. Clin Endocrinol (Oxf). 2009;71:459–65. 19222487 10.1111/j.1365-2265.2009.03545.x

[14] Res U Res P Kastelic D. Birth after treatment of a male with seminoma and azoospermia with cryopreserved-thawed testicular tissue. Hum Reprod. 2000;15:861–4. 10739833 10.1093/humrep/15.4.861

[15] Köhn FM Schroeder-Printzen I Weidner W. Testicular sperm extraction in a patient with metachronous bilateral testicular cancer. Hum Reprod. 2001;16:2343–6. 11679517 10.1093/humrep/16.11.2343

[16] Delouya G Baazeem A Boman JM. Identification of spermatozoa in archived testicular cancer specimens: implications for bench side sperm retrieval at orchiectomy. Urology. 2010;75:1436–40. 20035981 10.1016/j.urology.2009.10.039

[17] Choy JT Wiser HJ Bell SW. Predictors of spermatogenesis in orchiectomy specimens. Urology. 2013;81:288–92. 23374785 10.1016/j.urology.2012.10.038

[18] Brydøy M Fosså SD Klepp O. Norwegian Urology Cancer Group III study group. Paternity and testicular function among testicular cancer survivors treated with two to four cycles of cisplatin-based chemotherapy. Eur Urol. 2010;58:134–40. 20395037 10.1016/j.eururo.2010.03.041

[19] Bujan L Walschaerts M Moinard N. Impact of chemotherapy and radiotherapy for testicular germ cell tumors on spermatogenesis and sperm DNA: a multicenter prospective study from the CECOS network. Fertil Steril. 2013;100:673–80. 23755953 10.1016/j.fertnstert.2013.05.018

[20] Tomlinson M Kohut T Hopkisson J. Routine sperm banking for testicular cancer patients should be performed both before and after orchidectomy. J Clin Urol. 2013;6:171–6.

[21] Ogouma L Berthaut I Lévy R. Testicular sperm extraction (TESE) outcomes in the context of malignant disease: a systematic review. Asian J Androl. 2022;24:584–90. 35259785 10.4103/aja2021129 PMC9809488

[22] Suzuki K Shin T Shimomura Y. Spermatogenesis in tumor-bearing testes in germ cell testicular cancer patients. Hum Reprod. 2015;30:2853–8. 26428212 10.1093/humrep/dev250

[23] Shoshany O Shtabholtz Y Schreter E. Predictors of spermatogenesis in radical orchiectomy specimen and potential implications for patients with testicular cancer. Fertil Steril. 2016;106:70–4. 27005273 10.1016/j.fertnstert.2016.03.012

[24] Blecher GA Chung E Katz D. Onco-testicular sperm extraction (oncoTESE): a contemporary concept review and report of Australian sperm retrieval rates and fertility outcomes. Urology. 2022;160:109–16. 34813838 10.1016/j.urology.2021.10.031

[25] Cirigliano L Falcone M Gül M. Onco-TESE (Testicular Sperm Extraction): insights from a tertiary center and comprehensive literature analysis. Medicina (Kaunas). 2023;59:1226. 37512038 10.3390/medicina59071226 PMC10386487

[26] Fosså SD Chen J Schonfeld SJ. Risk of contralateral testicular cancer: a population-based study of 29,515 U.S. men. J Natl Cancer Inst. 2005;97:1056–66. 16030303 10.1093/jnci/dji185

[27] Klatte T de Martino M Arensmeier K. Management and outcome of bilateral testicular germ cell tumors: a 25-year single center experience. Int J Urol. 2008;15:821–6. 18657202 10.1111/j.1442-2042.2008.02107.x

[28] Carrasquillo R Sávio LF Venkatramani V. Using microscope for onco-testicular sperm extraction for bilateral testis tumors. Fertil Steril. 2018;109:745. 29653719 10.1016/j.fertnstert.2018.01.016

[29] Descombe L Chauleur C Gentil-Perret A. Testicular sperm extraction in a single cancerous testicle in patients with azoospermia: a case report. Fertil Steril. 2008;90:443.e1–4. 10.1016/j.fertnstert.2007.07.1308 18023440

[30] Roque M Sampaio M Salles PG. Onco-testicular sperm extraction: birth of a healthy baby after fertility preservation in synchronous bilateral testicular cancer and azoospermia. Andrologia. 2015;47:482–5. 24846759 10.1111/and.12292

[31] Carmignani L Gadda F Gazzano G. Testicular sperm extraction in cancerous testicle in patients with azoospermia: a case report. Hum Reprod. 2007;22:1068–72. 17172283 10.1093/humrep/del468

[32] Hamano I Hatakeyama S Nakamura R. Onco-testicular sperm extraction (Onco-TESE) from a single testis with metachronous bilateral testicular cancer: a case report. Basic Clin Androl. 2018;28:1. 29416867 10.1186/s12610-018-0066-2 PMC5785797

